# Seroprevalence of ANTI-SARS-CoV-2 antibodies in patients with inflammatory bowel disease

**DOI:** 10.1038/s41598-023-33402-w

**Published:** 2023-04-29

**Authors:** M. D. Martin-Arranz, L. García-Ramírez, M. Hernandez-Perez, D. Montero Vega, E. Martín-Arranz, M. Sánchez-Azofra, J. Poza Cordon, J. L. Rueda Garcia, J. Noci Belda, T. Verges Martínez-Meco, P. Blanco San Miguel, C. Suarez Ferrer

**Affiliations:** 1 Gastroenterology Department. IBD Unit., La Paz University Hospital, Madrid, Spain; 2 Faculty of Medicine, Universidad Autónoma, Madrid, Spain; 3 Hospital La Paz Institute for Health Research, La Paz University Hospital, Madrid, Spain; 4 Microbiology Department, La Paz University Hospital, Madrid, Spain

**Keywords:** Microbiology, Gastroenterology

## Abstract

Patients with inflammatory bowel disease (IBD) treated with biologic and/or immunosuppressant drugs are at increased risk for opportunistic infections. Seroprevalence studies can confirm the diagnosis of SARS-CoV-2 infections as well as the associated risk factors. This is a descriptive study which primary endpoints were to highlight the prevalence of SARS-CoV-2 antibodies in a cohort of IBD patients in March 2021, and to analyze seroconversion in patients with known COVID-19 infection and its relationship with IBD treatments. Patients filled in a questionnaire about symptoms of COVID-19 infection and clinical information about their IBD. All included patients were tested for SARS-CoV-2 antibodies. 392 patients were included. Among patients with clinical infection, 69 patients (17,65%) were IgG-positive, 286 (73,15%) IgG-negative and 36 (9,21%) indeterminate. In relation to seroconversion among patients under biologic treatment, 13 patients of the 23 with a previous positive CRP developed antibodies (56.5%). However, when the influence of immunosuppressive treatment on the probability of developing antibodies was analyzed, no significant differences were seen between those patients with or without treatment (77.8% vs. 77.1%, *p* = 0.96). In our cohort of IBD patients, after one year of pandemic, there were 18.64% IgG positive patients, a higher prevalence than the general population (15.7%).

## Introduction

The COVID-19 pandemic was declared by the WHO on 11 March 2020, following the first cases of SARS-CoV-2 coronavirus pneumonia reported from Wuhan (China) in December 2019. The most frequent clinical manifestations are fever, cough and dyspnoea, which may be complicated by acute respiratory distress syndrome, multi-organ failure or death. However, digestive symptoms (diarrhoea, vomiting or abdominal pain) are described in up to 20% of patients^1^.

This infectious disease represents a key challenge for gastroenterologists and patients with inflammatory bowel disease (IBD), a potential risk group that has required special attention during the pandemic. Given that altered immune response plays a key role in the pathogenesis of IBD and that the vast majority of drugs used modulate immune system function, IBD patients were considered to be at high risk for SARS-CoV-2 infection and severe disease^2^. However, current data do not appear to demonstrate increased risk of infection or severity in patients with IBD relative to the general population, except for those on corticosteroid therapy^3^. Even these patients present a mild course of infection, with low incidence of hospital admission, and a low fatality rate^3,4^. Active disease, age, and comorbidities are risk factors for COVID-19 mortality in patients with IBD^5^.

Under IOIBD supervision, an international registry, SECURE-IBD, was created and has been tracking reported cases since 15 March 2020. As of 15 October, 2021, 6,635 cases have been reported, of which only 144 required artificial ventilation, and 105 deaths in patients with IBD in association with COVID-19 disease.

Immunologically, the Spike (S) protein is a key immune target during coronavirus infections. This protein is closely associated with the production of neutralizing antibodies and protective immunity^6–8^*.* Protein S is responsible for the interaction of SARS-CoV-2 with host cells through ACE2 binding^9^. It can be divided into two regions, S1 and S2. The S1 region contains, in its second domain, the receptor binding domain (RBD)^10^. This makes serological testing a useful tool for identifying patients who have contracted the infection.

Figueiredo-Campos et al. demonstrated that antibodies against SARS-CoV-2 protein S and its RBD domain are easily detectable in most cases, even in patients receiving immunosuppressants or antiretroviral therapy. Under a classical immune response, antibodies for SARS-CoV-2 in the blood reach a peak at around week three post-infection, and although antibody levels are reduced, IgG antibodies remain detectable and show neutralising activity for at least 6 months after infection^11^.

It is known that the serological response to vaccination may be lower in immunocompromised patients^12^, and in the specific case of IBD, several studies have shown that the response to hepatitis B or anti-pneumococcal vaccination is lower in patients treated with immunosuppressants and/or biological therapy^13–15^.

The aim of our study was to define, in patients with IBD:

- The seroprevalence of IgG-SARS-CoV-2 one year after the beginning of the pandemic in an area at high risk of infection (Madrid, Spain)
- Seroconversion in patients with known COVID-19 infection and its relationship to drugs used in the treatment of IBD.

## Material and methods

This is a single-centre, descriptive study of a cohort of patients with pre-exisiting diagnosis of IBD, recruited between 26 February and 26 March 2021, one year after the start of the pandemic in our country (the first case in Madrid, Spain, was diagnosed on 25 February 2020).

Patients with IBD under follow-up in our unit and with scheduled on-site visits were included. Patients who had received any dose of COVID-19 vaccine were not included. The patients included completed a detailed questionnaire on symptoms indicative of SARS-CoV-2 infection, demographic data, and clinical information about their IBD (Appendix 1).

The WHO definition of cases was used as reference^16^, which evaluates the results of PCR, antigen determination and symptomatology to define confirmed, probable and suspected cases. A confirmed case is: a person who has tested positive on a nucleic acid amplification test (NAAT), a person who has tested positive on a rapid antigen detection test for SARS-COV and meets clinical or radiological criteria for a probable or suspected case, or an asymptomatic person who has tested positive on a rapid antigen detection test and has had contact with a probable or confirmed case.

All included patients underwent qualitative serology by two Chemiluminescent immunoassay (CLIA) techniques: Siemens (Atellica), which detects total antibodies against S and Vircell (Virclia), which detects IgG against S and N. For Siemens, the cut-off point is one. Above this value it was considered positive, and below, negative. For VirClia, below 1.4 is negative; between 1.4 and 1.6 is a doubtful result, and above 1.6 is positive. If the Siemens test was positive, it was considered as IgG positive. If the Siemens result was low positive or negative, the VirClia test was performed. If both determinations coincided, it was given as IgG positive or negative; but if both results did not coincide, the result was indeterminate.

It was decided to perform different determinations previously checked in the Microbiology Department due to the disparity in the findings reflected in some studies^17^.

### Ethical considerations

The study was evaluated by the Ethics Committee of the La Paz University Hospital, Madrid (ID code PI-4637). All patients included signed an informed consent form in order to participate in the study. The study protocol conforms to the ethical guidelines of the 1964 Declaration of Helsinki.

### Statistical analysis

Since this is a descriptive study that does not include contrast of hypothesis, no prior calculation of the sample size is necessary.

A descriptive analysis of baseline and IBD-related characteristics was performed. For continuous variables, the mean and standard deviation were calculated; for categorical variables, percentages and 95% confidence intervals were calculated. Provided that the variables had a normal distribution (verified by the Shapiro Wilk test), categorical variables were compared using the ^2^ test, and quantitative variables were compared using the Student's t-test. Otherwise, the corresponding non-parametric test was applied. A value of *p* < 0.05 was considered statistically significant.

## Results

A total of 392 patients were included, the baseline characteristics are summarized in Table 1 and those related to their IBD in Table 2. 8 patients were previously excluded because they had already received a dose of COVID-19 vaccine.

**Table 1:** Baseline characteristics.

Variable	
Sex	Male 204 (52%)
	Female 188 (48%)
Country of birth	Spain 360 (91%)
	Others 32 (9%)
Smoker	No 200 (53.3%)
	Yes 62 (16.5%)
	Ex 113 (30.2%)
Employment situation	Retired 86 (25%)
	Unemployed 39 (11.2%)
	In-person work 110 (32.3%)
	Partially remote work 47 (13.6%)
	Remote work 100% 60 (17.3%)
	Sick leave 2 (0.6%)
Comorbidity	No 301 (77.8%)
	Yes 86 (22.2%)
History of cancer	No 362 (92.35%)
	Yes 30 (7.65%)
Chronic kidney disease	No 380 (96.94%)
	Yes 12 (3.06%)
COPD	No 381 (97.19%)
	Yes 11 (2.81%)
Asthma	No 374 (95.9%)
	Yes 16 (4.1%)
Diabetes	No 365 (93.1%)
	Yes 27 (6.9%)
Obesity	No 359 (91.5%)
	Yes 33 (8.5%)
Cardiovascular disease	No 355 (91.7%)
	Yes 32 (8.3%)
HBP	No 323 (83.46%)
	Yes 64 (16.54%)

**Table 2:** IBD characteristics.

Previous surgeries	No 295 (75.45%) Yes 96 (24.55%)
IBD type	Crohn's disease 210 (53.57%)
	Ulcerative colitis 155 (39.54%)
	Indeterminate colitis 27 (6.9%)
IBD duration	Mean 4,6 years (SD 0,04)
EIMS	No 290 (74.36%)
	Yes:
	Peripheral spondyloarthropathy 32 (8.21%)
	Ankylopoietic spondylitis 8 (2.05%)
	Sacroilitis 8 (2.05%)
	Cutaneous 15 (3.05%)
	Ocular 0/5 (1.28%)
	Sclerosing cholangitis 3 (0.77%)
IBD therapy	Corticosteroids 9 (2.3%)
	Immunomodulators 47 (12.1%)
	Combined Treatment 201 (52%)

The average age of the patients included at the time of analysis was 49.23 years (SD: 15.4). Regarding the treatments followed by patients at the time of the start of the pandemic (March 2020): monotherapy with biologics was the most frequent (105 patients, 26.99%); followed by patients with combined treatment (biologic + immunosuppressant) (60 patients, 15.42%) and immunosuppressants in monotherapy (57 patients, 14.65%). 43% of patients were not under treatment with any of the above drugs. Regarding the type of biologic used by patients, 43% (62 patients) were on Infliximab, 21% (30 patients) on Adalimumab, 15% (22 patients) on Vedolizumab and 13.2% (19 patients) on Ustekinumab.

At the time of analysis (July 2021), the percentage of patients on biologics had increased compared to the beginning of the pandemic, with therapy that was alone or in combination with immunosuppressants (201 patients, 52%), while those on immunomodulatory therapy alone decreased to 12.1% (47 patients). Finally, 9 patients (2.3%) were on corticosteroid treatment. The distribution in terms of the use of the different biological drugs did not differ significantly from that shown for March 2020.

With respect to the serology extracted to assess the seroprevalence of IgG antibodies against SARS-CoV-2 in patients with IBD, 286 patients (73.15%) were negative, 69 patients (17.65%) were positive and in 36 patients (9.21%) the result was indeterminate.

Among the included patients, 80 patients (21%) had had a previous probable or confirmed SARS-CoV-2 infection, of whom 52 patients (66.7%) had a positive PCR. Of these, 27.8% (22 patients) had previous documented positive serology (IgG SARS-CoV-2).

The most frequent symptoms among these patients who had had the infection were fever (61.4%), general malaise (70.5%) and headache (64.1%). The duration of these symptoms averaged 17.04 days (SD: 21.16). In our experience, 19.23% of the sample (15 patients) required hospital admission due to COVID-19.

Among the 15 patients admitted, only 5 presented comorbidities. However, only 14 of our sample had comorbidity (HBP, DM, obesity, cardiovascular disease, COPD, asthma, chronic renal disease, personal history of cancer, etc.) and of these, 5 (35.7%) were admitted to hospital (*p* = 0.09). In the logistic regression analysis, HBP (OR 2.99, IC95% 0,3–21,9) and obesity (OR 4.15, IC95% 0,5–43,1) were shown to be significant.

Furthermore, it was not found that the percentage of patients requiring hospitalisation differed between those under treatment with biological and/or immunosuppressant drugs and others without these therapies (*p* = 0.16), with 12% of admissions being those with biological therapies.

The possibility of developing IgG antibodies against SARS-CoV-2 in patients with previous positive PCR was analysed. Among the 52 patients with documented positive PCR, only 34 patients (65.4%) had positive antibodies and 8 of them (15.3%) had indeterminate IgG titres (*p* = 0.53). Time between positive PCR and SARS-CoV-2 antibodies test was 169 days (median) (IQR: 85–329).

We obtained similar data when investigating the probability of developing antibodies in those patients with criteria of past infection (although not necessarily with a positive PCR test): 52 of the 80 patients (65%) had positive antibodies (and in 10 patients (12.5%) they were indeterminate), compared to 16 patients who had positive antibodies among those 305 (5.2%) with no history of COVID-19 infection, *p* = 0.0001.

One of the most important issues to analyse is the rate of seroconversion in patients who were on biologics or immunosuppressants.

When seroconversion was analysed among those patients under biological treatment, 13 patients of the 23 with previous positive PCR developed antibodies (56.5%), and in 4 cases the results were indeterminate (17.4%). However, when we analysed the influence of immunosuppressant treatment on the probability of developing antibodies among those patients with a previous positive PCR test, we identified that they were similar between those with or without treatment (77.8% vs 77.1%, *p* = 0.96).

We analysed the influence of biological treatment with anti-TNF on the possibility of developing antibodies, finding that among patients with a positive PCR test 54.5% (6/11 patients) developed antibodies when using anti-TNF drugs compared to 84% (20/33 patients) among those with any other treatment (*p* = 0.03).

**Figure 1:**
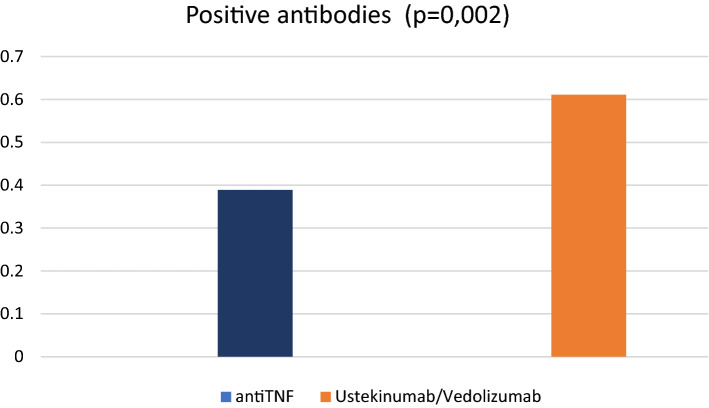
Percentage of antibody-positive patients on anti-TNF or Vedolizumab/Ustekinumab therapy.

Furthermore, when we compared the influence of the different types of biologic according to their therapeutic target, we identified that, overall, 38.9% (7/84 patients) of patients on anti-TNF had positive antibodies compared to 61.1% (11/37 patients) among those on Ustekinumab or Vedolizumab (*p* = 0.002) (Fig. 1).

Similarly, when we analysed seroconversion rates among patients with a previous positive PCR test, we found that it was 54.5% (6/11 patients) in the anti-TNF group versus 85.1% (6/7 patients) in the Vedolizumab or Ustekinumab group (*p* = 0.17).

Finally, we did not find that other baseline characteristics, such as sex, patient age or type of IBD, of the included patients were statistically significantly associated with antibody formation. However, when we analysed the influence of the patients' work situation during the pandemic, we found that of those with in-person or semi in-person jobs 24.1% (35/145 patients) had positive antibodies compared to 16% (34/210 patients) who did not need to go to their workplace either because they were working remotely, were retired or unemployed (*p* = 0.05).

It should be noted that, of the 86 patients with associated comorbidity, 16 patients (18.6%) developed antibodies compared to 29% without the presence of comorbidity (*p* = 0.045).

## Discussion

In our cohort of patients, 17.65% of patients showed IgG-SARS-CoV-2 at one year after the onset of the pandemic. In Spain, the Ministry of Health conducted a population-based seroprevalence study using a rapid antibody test that estimated a positivity rate of 11.7% (CI 10.3–13.3) for Madrid in June 2020^18^. This difference may be due to the different types of assays used, and the difference in the time of performance, but it is consistent with a seroprevalence in Madrid higher than 10% (significantly higher than other geographical areas of Spain).

It is important to note that over 50% of the patients included in the study were under biological/immunosuppressant treatment or both.

Furthermore, we observed that in our routine clinical practice, and following the recommendations of the different scientific societies, we did not suspend immunosuppressant or biological therapies in a generalised manner;^19,20^ in fact, the number of patients treated with biologics increased, avoiding the use of corticosteroids, a drug that has been associated with greater severity of the disease^3^.

One of the most relevant data of the study is that only 65% of the patients with data of probable or previously confirmed infection had antibodies at the time of the study, this figure being much lower than that reported in the general population, which exceeds 90% of seroconversion^21,22^. However, given the cross-sectional nature of the study, this data may be due to a loss of antibodies months after infection or due to the lack of antibody development in our patient population. This percentage is even lower in patients treated with biologics, although when compared with the percentage in patients not exposed to these treatments, statistical significance is not reached. Antibody concentrations have been shown to remain attenuated in patients treated with infliximab. In these patients, reinfection has been more frequent and has occurred earlier than in patients with other treatments^23^.

When analysing the seroconversion data in relation to the biological drugs used by the patients, it is relevant to point out that patients on anti-TNF treatment have a lower percentage of positive serology compared to other biologics (Ustekinumab and Vedolizumab), and this fact is maintained among those with known previous infection. This finding is consistent with other published work^24^.

Finally, we found no influence of other variables such as type of disease, sex or age on the presence of antibodies, but we did find a possible greater exposure in those who continued to attend their place of work in person compared to those who remained at home.

The limitations of the study include the fact that during the first wave of the pandemic (March to May 2020), the emergency health situation meant that some of the cases did not have microbiological confirmation, so the diagnosis was made solely on the basis of clinical/radiological criteria following the WHO definition. Moreover, since this is a cross-sectional study, it is difficult to differentiate which patients with past SARS-CoV-2 infection did not develop antibodies and which developed antibodies after primary infection and subsequently lost them.

## Conclusions

The seroprevalence of antibodies to SARS-CoV-2 is 17.65% one year after the beginning of the pandemic in a region with a high prevalence of infection. In our cohort, the presence of antibodies is less likely among those treated with anti-TNF than with other biologics and, overall, there is less seroconversion after known infection than in the general population. These findings may impact future protective measures and vaccination in this patient population.

The datasets generated during and/or analysed during the current study are available from the corresponding author on reasonable request.

## Supplementary Information

Supplementary Information.
