# Supplementary material for: Seroprevalence of ANTI-SARS-CoV-2 antibodies in patients with inflammatory bowel disease
Source: Sci Rep. 2023 Apr 29;13:7044. doi: 10.1038/s41598-023-33402-w (PMC10148621; doi:10.1038/s41598-023-33402-w)
Supplement: Supplementary file 1 — Supplementary Information. [file 41598_2023_33402_MOESM1_ESM.pdf]

STUDY OF SEROPREVALENCE OF COVID-19 INFECTION IN PATIENTS WITH INFLAMMATORY BOWEL DISEASE

First and last name:

Actual treatment for IBD:

Associated diseases:

PATHOLOGY	YES	NO	DATE OF DIAGNOSIS
CANCER			
CHRONIC KIDNEY DISEASE			
CHRONIC OBSTRUCTIVE PULMONARY DISEASE			
ASMA			
DIABETES			
OBESITY			
CARDIOVASCULAR DISEASE			
ARTERIAL HYPERTENSION			
ORGAN TRANSPLANTATION			

Employment status as of September 2020:

- ☐ Retired
- ☐ Unemployed
- ☐ On-site work
- ☐ Semi-attendance work
- ☐ Full teleworking

Have you been diagnosed with COVID-19 infection?

Diagnosis: ☐ PCR ☐ IgG

Date of diagnosis:

Symptom onset date (if any):

Approximate duration of symptoms:

Did you require hospitalization?

SYMPTOM	YES	NO
FEVER		
CEPHALEA		
GENERAL DISCOMFORT		
MIALGIAS		
RINORREA		
DYSGEUSIA		
ANOSMIA		
COUGH		
PRODUCTIVE COUGH		
THORAX PAIN		
ODYNOPHAGIA		
COSTAL PAIN		
DYSNEA		
